# Study on basic and clinical application of Shufeng Jiedu Capsule in treating respiratory tract infection

**DOI:** 10.1186/s13020-023-00749-1

**Published:** 2023-04-25

**Authors:** Yanqi Han, Jun Xu, Qiang Zhu, Li Yang, Yitao Wang, Hua Luo, Tiejun Zhang

**Affiliations:** 1 State Key Laboratory of Drug Delivery Technology and Pharmacokinetics, Tianjin Institute of Pharmaceutical Research Co., Ltd, Tianjin, 300462 China; 2 Macau Centre for Research and Development in Chinese Medicine, State Key Laboratory of Quality Research in Chinese Medicine, Institute of Chinese Medical Sciences, University of Macau, Macau SAR, 999078 China; 3 Anhui Jiren Pharmaceutical Co., Ltd, Bozhou, 236800 Anhui China

**Keywords:** Big brand traditional Chinese medicine, Shufeng Jiedu Capsule, Secondary development of TCM, Respiratory tract infections, Systematical research

## Abstract

Shufeng Jiedu Capsule (SFJDC), composed of eight herbs, is a big brand traditional Chinese medicine (TCM) for the treatment of different respiratory tract infectious diseases with good clinical efficacy and few side effects. It is clinically applied to acute upper respiratory tract infection(URI), influenza, acute exacerbation of chronic obstructive pulmonary disease (AECOPD), community-acquired pneumonia(CAP) and other diseases, due to its antibacterial, antiviral, anti-inflammatory, immunoregulatory and antipyretic activities. In particular, it has shown good clinical effects for COVID-19, and was included in the fourth to tenth editions of the ‘Diagnosis and Treatment Protocol for COVID-19 (Trial)’ by the National Health Commission. In recent years, studies on the secondary development which focus on the basic and clinical application of SFJDC have been widely reported. In this paper, chemical components, pharmacodynamic material basis, mechanisms, compatibility rule and clinical application were systematically summarized, in order to provide theoretical and experimental basis for further research and clinical application of SFJDC.

## Introduction

Shufeng Jiedu Capsule (SFJDC) is a big brand traditional Chinese medicine (TCM) for the treatment of respiratory tract infection. It comprises eight herbs, including Polygoni cuspidati rhizoma (Huzhang), Forsythiae fructus (Lianqiao), Isatidis radix (Banlangen), Bupleuri radix (Chaihu), Patriniae herba (Baijiangcao), Verbenae herba (Mabiancao), Phragmitis rhizoma (Lugen), Glycyrrhizae radix (Gancao). SFJDC has the effect of clearing heat and detoxifying, dispelling wind and relieving exterior, which has been used to treat acute upper respiratory tract infection (URI) with external wind-heat syndrome.Indicative symptoms are fever, aversion to cold, sore throat, headache, nasal congestion, runny nose and cough [1–3]. Clinical application studies have shown that SFJDC can also treat community-acquired pneumonia, chronic obstructive pulmonary disease(COPD), and other diseases [4–8]. It is recommended as the treatment guideline and recommended medication for 12 major diseases, including ‘TCM Diagnosis and Treatment Protocol for Wind-warm Lung-heat Disease (Viral Pneumonia) (Mild Cases)’ (2017), ‘Diagnosis and Treatment Protocol for exogenous fever (URI)’ (2017), and is also included in the ‘National Reimbursement Drug List’ and ‘National Essential Medicine List’. During the prevention and control of the COVID-19 pandemic, SFJDC is the recommended drug for the fourth to tenth editions of the “Diagnosis and Treatment Protocol for COVID-19 (Trial)” issued by National Health Commission. In this paper, the basic research and clinical application of SFJDC in the treatment of respiratory tract infectious diseases were reviewed to provide an important theoretical and experimental basis for the clinical application of this variety.

## Systematical identification and transferring rules of chemical components

**Fig. 1:**
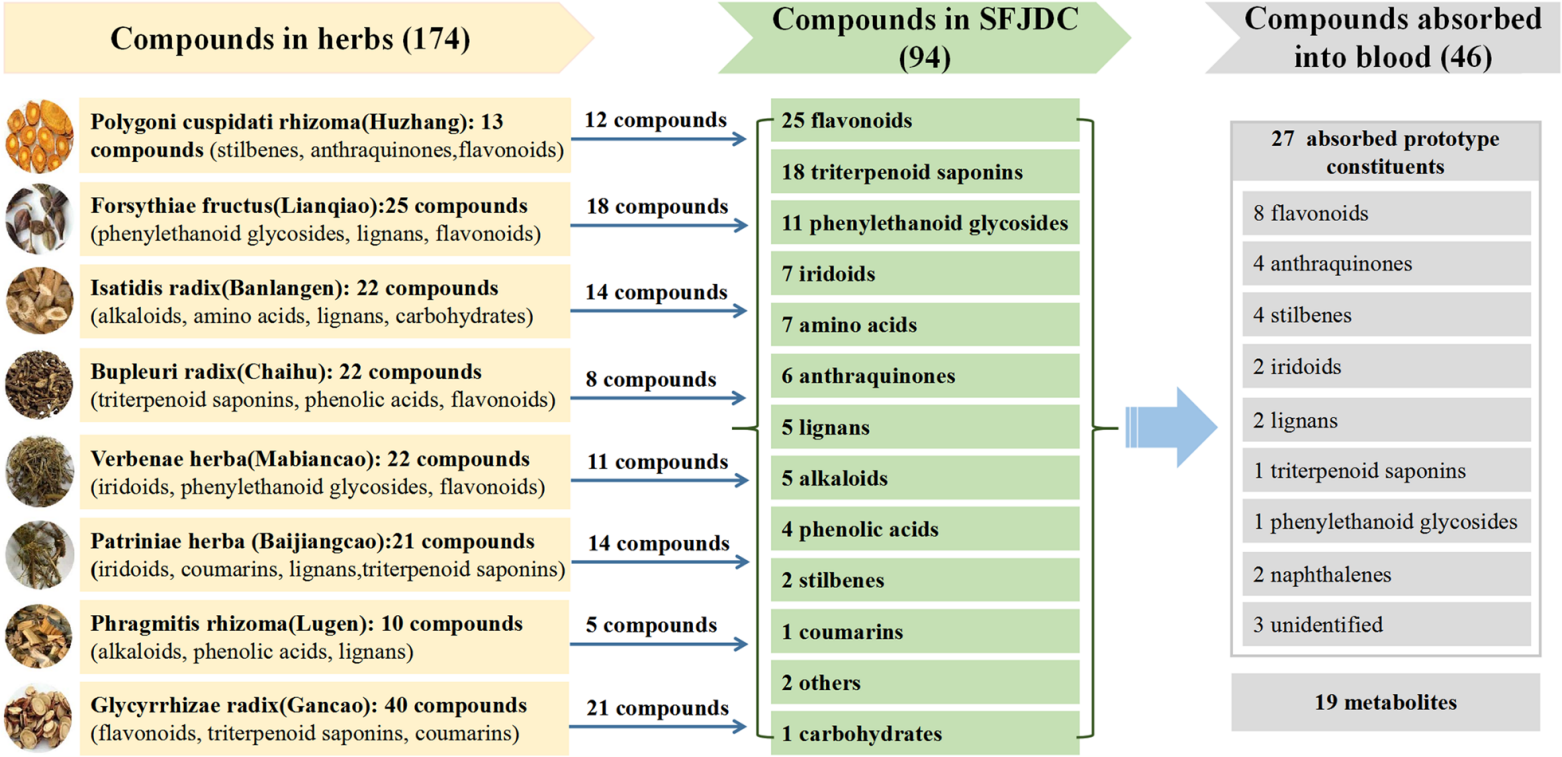
Summary of ‘herb-prescription-blood’ components of SFJDC

The chemical components of raw materials, preparations, blood components and metabolites of SFJDC were characterized and identified (summarized in Fig. 1), and the chemical basis and transfer rules of SFJDC were clarified. It provides the premise and basis for explaining the pharmacodynamic material basis, mechanism and formulating scientific quality control methods and quality control systems.

The chemical components of eight herbs were identified by Ultra-performance liquid chromatography quadrupole time-of-fight mass spectrometry (UPLC-Q-TOF–MS/MS) [9–12]. A total of 174 components were identified, of which 13 compounds were identified from Polygoni cuspidati rhizoma, 25 compounds were identified from Forsythiae fructus, 22 compounds were identified from Isatidis radix, 22 compounds were identified from Bupleuri radix, 22 compounds were identified from Verbenae herba, 21 compounds were identified from Patriniae herba, 10 compounds were identified from Phragmitis rhizoma, 40 compounds were identified from Glycyrrhizae radix.

**Table 1:** Summary of pharmacodynamics, mechanism and pharmacodynamic material basis of SFJDC

Activity	Pharmacodynamics and mechanism	Pharmacodynamic material basis
Antibacterial and antiviral	Inhibiting *Staphylococcus aureus, Staphylococcus sp., Staphylococcus epidermidis, Streptococcus spp., Escherichia coli, Pseudomonas aeruginosa, Streptococcus pneumonia, Proteus sp., Neisseria gonorrhoeae, Candida albicans, Shigella dysenteriae* and *β-Hemolytic streptococcus.*^[21.22]^; Inhibiting H1N1 (FM1strain, PR8 strain, Jiangxi Xiushui strain, B10 strain, B59 strain), HSV-1, HSV-2, RSV, parainfluenza virus (Sendai strain), CBV3, CBV4, CBV5 [22–25, 27]; Regulating miR-155/JAK1-STAT1 [26] and MAPK/NF-κB pathways [18]; Reducing IL-1β, IL-18 levels in serum and BALF, reducing NLRP3-associated components and viral titers in lung homogenates [6]; Decreasing TNF-α, IL-6, IL-10 and IFN-γ in the lungs, increasing the amount of CD4 + and CD8 + cells in the blood [28]; Binding with SARS-CoV-2, ACE2, TNF, STAT1, CCL2, RELA, MAPK1 to exert therapeutic effect on COVID-19 [29–33]	Licochalcone a [31], quercetin, luteolin, kaempferol, wogonin, baicalein and polydatin [28–30]
Anti-inflammatory	Inhibiting the levels of IL-1β, TNF-α, P-selectin, TGF-β, keratinocyte-derived chemokine, c-Jun/AP-1, NF-κB mRNA in lung tissue; suppressing MAPK/NF-κB pathway [34, 35]; Regulating PI3K/Akt and ERK pathways [15, 17]; Regulating GPR18 [37], A2AAR [38]; Mediating autophagy and apoptosis, suppressing inflammatory factors release [39]; Inhibiting IL-1α, IL-1β, IL-2, IL-4, IL-10, IL-12(p70), IL-13, IL-17,G-CSF, GM-CSF, IFN-γ, KC, TNF-α in LPS-induced macrophage [40]	Forsythoside E, forsythoside A, isoforsythiaside A, verbascoside, hastatoside, verbenalin, forsythin, 3-hydroxyglabrol, vitexin, emodin, phillyrin, polydatin, rhein, emodin-8-monomethylether, 6-hydroxyaloeemodin, physcion, deoxyglabrolide, liquiritigenin, adoxosidic acid [15, 17–19]
Immunoregulatory	Reducing B cells(%), CD8 + (%) and IL-1α, IL-1β, IL-2, IL-10, TNF-α, IFN-α, IFN-γ, IgM, IgG levels in serum, reducing the weight of thymus, spleen and lung, increasing CD4 + /CD8 + , NK(%) [41]; Increasing GSH-Px, SOD, inhibiting MDA and inflammatory factors [42]; Adjusting the ratio of CD3 + , CD4 + /CD8 + of serum T lymphocyte subsets [43]; Interfering with several key proteins such as HRAS, MAP2K1, AKT1, MME, and PTPN1 [44]	Dihydrodylate glycoside, caffeic acid, liquiritin, emodin, phillyrin, verbenalin, 7-methoxy isorhamnetin, kaemferol-3-*O*-rhamnoside [44]
Antipyretic	Reduce body temperature [45]; Reducing PGE2, TNF-α, IL-1α, IL-1β, IL-6, MIP-1α, cAMP, cAMP/cGMP levels in serum and hypothalamus; Declining Na^+^, K^+^-ATPase, NOS, NO levels in hypothalamus [46]; Enhancing AVP content in hypothalamus; Reducing TLR4, NF-κB p65, IkBα and COX2 expression in hypothalamus [47]	Glycyrrhizic acid, dihydropatrinoside, saikosaponin D, forsythoside E, forsythoside I, verbascoside, isoverbascoside, forsythin, pinoresinol-4-*O*-*β*-D-glucoside, emodin, emodin-1-*O*-glucoside, rhein, trans-polydatin, liquiritigenin, liquiritin, 7-methoxyisorhamnetin [20]

A total of 96 ion flow chromatographic peaks from SFJDC were found by HPLC-Q-TOF–MS/MS, and 94 compounds were identified, including 7 amino acids, 1 carbohydrate component, 7 iridoids, 11 phenylethanoid glycosides, 2 stilbenes, 25 flavonoids, 5 lignans, 6 anthraquinones, 18 triterpenoid saponins, 1 coumarins, 4 phenolic acids, 5 alkaloids and 2 other small molecule compounds. The sources of all compounds were assigned, of which 12 from Polygoni cuspidati rhizoma, 18 from Forsythiae fructus, 14 from Isatidis radix, 8 from Bupleuri radix, 14 from Patriniae herba, 11 from Verbenae herba, 5 from Phragmitis rhizoma, 21 from Glycyrrhizae radix [13–15].

Furthermore, UPLC-Q-TOF–MS/MS was used to identify the absorption prototype components and their metabolites in rats plasma after oral administration of SFJDC [16]. A total of 46 exogenous compounds related to SFJDC were identified in rats plasma, including 27 absorption prototype drug components ( 8 flavonoids, 4 anthraquinones, 4 stilbenes, 2 iridoids, 2 lignans, 2 naphthalenes, 1 phenylethanoid glycoside, 1 triterpenoid saponin and 3 other compounds) and 19 metabolites. The absorption prototype components and their metabolites detected in the plasma of the administered rats may be the potential real active components of the formula and are directly related to the pharmacological effects of SFJDC.

## Study on the pharmacodynamic material basis

Anti-inflammatory is an important mechanism of SFJDC in treating acute upper respiratory tract infection. Tiejun Zhang et al. [13, 17] took inflammation as the breakthrough point and used UPLC-Q-TOF–MS/MS to integrate the screening system of nuclear factor kappa-B(NF-κB) dual-luciferase reporter gene system to quickly and accurately screen and identify the potential anti-inflammatory active ingredients in SFJDC, by which 10 active compounds containing forsythoside E, forsythoside A, isoforsythiaside A, verbascoside, hastatoside, verbenalin, forsythin, 3-hydroxyglabrol, vitexin, emodin were determined. Yanmei Li et al. [15] reported that, 14 compounds in SFJDC including phillyrin, verbenalin, hastatoside, verbascoside, forsythoside E, polydatin, rhein, emodin-8-monomethylether, 6-hydroxyaloeemodin, physcion, emodin, deoxyglabrolide, liquiritigenin, adoxosidic acid, were related to the anti-inflammatory ERK signaling pathway and further inferred that phillyrin, emodin and verbenalin may act synergistically against inflammation. Another study [18] screened potential antivirus compounds in SFJDC by a bioactivity-integrated method of UPLC-Q-TOF–MS/MS combined with methyl thiazolyl tetrazolium assay on the H1N1-infected RAW264.7 cell models and 3 compounds (forsythoside E, verbenalin, and emodin) exerted the advantages of protective effects in cell vitality during H1N1 infection.

Yanqi Han et al. [19, 20] screened and determined the pharmacodynamic material basis of SFJDC from two aspects of ‘dispelling wind and relieving exterior (DWRE)’ and ‘clearing heat and detoxifying (CHD)’ through the ‘spectrum-effect’ analysis method. Eight herbs of SFJDC were proportioned by using uniform design and 22 combinations of SFJDC were selected as the research objects. The in vitro pharmacodynamic models (such as acetylcholine receptor, LPS induced inflammation model) related to DWRE and CHD were selected. The LC–MS spectrum analysis and in vitro cell activity experiment of SFJDC with different combinations were carried out. The obtained spectrum data and in vitro activity data were integrated and analyzed by using artificial neural network analysis (ANN) and other mathematical statistics methods to establish the ‘spectrum-activity’ relationship, screening out the components closely related to diaphoresis and anti-inflammatory effects. The results showed that 15 compounds (glycyrrhizic acid, dihydropatrinoside, saikosaponin D, forsythoside E, forsythoside I, verbascoside, isoverbascoside, forsythin, pinoresinol-4-*O*-*β*-D-glucoside, emodin, emodin-1-*O*-glucoside, rhein, trans-polydatin, liquiritigenin, liquiritin, 7-methoxyisorhamnetin) identified by the M3 model may be the material basis of DWRE effect of SFJDC. The 13 compounds (verbenalin, liquiritigenin, isoliquiritin, apiosyl-isoliquiritigenin, pinoresinol-4-*O*-*β*-D-glucoside, trans-polydatin, hydroxyglycyrrhizic acid, saikosaponin D, emodin, rhein, verbascoside, forsythiaside A, forsythiaside E, cassianone-8-*O*-glucoside) identified by the anti-inflammatory model may be the material basis of the CHD effect of SFJDC.

## Study on pharmacodynamics and mechanism

SFJDC is mainly used to treat acute URIs such as the flu, swelling and pain in the throat, mumps, and strep throat with antibacterial, antiviral, anti-inflammatory, immunoregulatory, and antipyretic properties.

### Antibacterial and antiviral activity

Based on clinical investigations and basic research, SFJDC has a broad-spectrum antibacterial and antiviral activity. Antibacterial studies [21, 22] indicated that SFJDC has broad-spectrum antibacterial activity against bacteria including *Staphylococcus aureus, Staphylococcus sp., Staphylococcus epidermidis, Streptococcus spp., Escherichia coli, Pseudomonas aeruginosa, Streptococcus pneumonia, Proteus sp., Neisseria gonorrhoeae, Candida albicans, Shigella dysenteriae* and *β-Hemolytic streptococcus.* Its antibacterial activity was superior to Lianhua Qingwen capsule and Qingkailing granules (QKL). SFJDC could lower the mortality rate, reduce mortality, increase average survival time and increase the lifespan of mice dying due to a *Staphylococcus aureus* or *Streptococcus* infection.

The in vitro antiviral activity of SFJDC was analyzed by cytopathic effect inhibition method. The results showed that SFJDC had an inhibitory effect on influenza A virus H1N1 (FM1strain, PR8 strain, Jiangxi Xiushui strain, B10 strain, B59 strain), herpes simplex virus 1 (HSV-1), herpes simplex virus 2 (HSV-2), respiratory syncytial virus (RSV), parainfluenza virus (Sendai strain), Coxsackievirus B4 (CBV4) and CBV5 [22, 23]. In vivo experiments showed that SFJDC could relieve pneumonia symptoms of immunocompromised or normal mice induced by influenza virus FM1 and PR8 of H1N1, significantly reduce pulmonary index, decrease death rate and prolong survival days, showing markedly therapeutic efficacy [24, 25]. Other studies illuminated that SFJDC attenuated pneumonia induced by influenza A virus H1N1 through miR-155/JAK1-STAT1 signaling pathway in mice [26] and Type I interferon(IFN), MAPK/NF-κB signaling pathways may also involve in the anti-H1N1 infection effects of SFJDC [18]. In another in vivo study, SFJDC combined with oseltamivir significantly attenuated influenza A virus-induced lung damage by reducing interleukin-1 β (IL-1β) and IL-18 levels in serum and bronchoalveolar lavage fluid (BALF), and reduced the expression levels of NOD-like receptor thermal protein domain associated protein 3(NLRP3)-associated components and viral titers in lung homogenates of rats [6]. In the mouse models infected with RSV, HSV-1 and CBV3, SFJDC reduced the lung index of RSV infected mice in a dose dependent manner, which slightly inferior to ribavirin, but significantly superior to QKL, and SFJDC also increased the survival and life extension rates of mice infected with RSV, HSV-1 and CBV3 [27]. Moreover, SFJDC significantly reduced the viral load in the lungs of mice infected with HCoV-229E, decreased the inflammatory factors tumor necrosis factor-α(TNF-α), IL-6, IL-10 and IFN-γ in the lungs, and increased the amount of CD4 + and CD8 + cells in the blood [28]. Network pharmacology experiments revealed that the active components of SFJDC,such as quercetin, luteolin, kaempferol, wogonin, and baicalein may regulate biological pathways related to inflammation, immune response, cell proliferation, apoptosis and migration by binding with SARS-CoV-2, ACE2, TNF, STAT1, CCL2, RELA, MAPK1, and others, to exert therapeutic effect on COVID-19 [29–33]. The broad-spectrum antiviral effect of SFJDC provides a theoretical basis for its clinical application, and is a potential therapeutic drug for new viral infections of respiratory diseases.

### Anti-inflammatory activity

Several studies have demonstrated that SFJDC can effectively relieve inflammation during acute lung injury (ALI). Zhengang Tao et al. [34, 35] investigated the protective effect of SFJDC on LPS-induced ALI rats. The results showed that, SFJDC could reduce the reaction of LPS-induced lung inflammation by inhibiting the levels of IL-1β, TNF-α, P-selectin, transforming growth factor-β, keratinocyte-derived chemokine, c-Jun/AP-1, NF-κB mRNA in lung tissue, which concluded that, SFJDC alleviated acute lung injury by suppressing the MAPK/NF-κB signaling pathway. Qualitative and label-free quantitative proteomics, RNA sequencing were conducted to explored the protective and therapeutic mechanisms of SFJDC. According to the results, it has speculated that SFJDC exerted anti-inflammatory effects by regulating PI3K/Akt and ERK signaling pathways [15, 17]. The effective compound verbenoside could effectively regulate the G protein-coupled receptor 18 (GPR18) and improve the lung injury induced by *Pseudomonas aeruginosa* in mice [36]. Junnan Cai et al. [37] conducted animal and cell studies to probe the role and mechanism of SFJDC in ALI induced by LPS. They found that SFJDC attenuated ALI by inhibiting the inflammatory response and cell apoptosis. The mechanism might be related to up-regulate A2AAR, increase cyclic adenosine monophosphate (cAMP) and inhibit phosphorylation of NF-κB. Similarly, lung injury in LPS-infected ALI rats could be protected by SFJDC by alleviating the inflammation response via NRF2-associated antioxidant pathway [38]. Jinyu Mei et al. [39] revealed that SFJDC alleviated ovalbumin-induced allergic rhinitis in a rat model by mediating levels of autophagy and apoptosis and by suppressing the release of inflammatory factors. In vitro experiments showed that, the anti-inflammatory mechanism of SFJDC might be related to inhibition of inflammatory cytokine release, including IL-1α, IL-1β, IL-2, IL-4, IL-10, IL-12(p70), IL-13, IL-17, granulocyte colony-stimulating factor(G-CSF), granulocyte–macrophage colony-stimulating factor(GM-CSF), IFN-γ, keratinocyte cytokines, TNF-α, in LPS-induced macrophage [40].

### Immunoregulatory activity

Li Ma et al. [41] used the pneumonia model caused by *Streptococcus pneumoniae* to observe the effect of SFJDC on immune response related factors. The results showed that SFJDC could significantly reduce the peripheral blood B cells(%), CD8 + (%) and serum IL-1α, IL-1β, IL-2, IL-10, TNF-α, IFN-α, IFN-γ, immunoglobulin M (IgM) and IgG levels, reduce the weight of thymus, spleen and lung, and increase the peripheral blood CD4 + /CD8 + and natural killer cells (NK) (%). Jing Shi et al. [42] found that SFJDC could significantly repair oxidative stress injury in COPD mice by increasing glutathione peroxidase (GSH-Px) content, increasing SOD activity, inhibiting the release of malondialdehyde(MDA) and inflammatory factors. It could reduce the pathological damage of lung tissue by enhancing the immune function of mice, which was helpful to promote the recovery of lung function. Feihu Wu et al. [43] reported that SFJDC had a significant therapeutic effect on acute pharyngitis rats and the mechanism may be that it had immunity-regulating effect by adjusting the ratio of CD3 + , CD4 + /CD8 + of serum T lymphocyte subsets. Network pharmacology trails indicated that SFJDC interfered with multiple biological processes related to anti-inflammation and immunoregulation by acting on several key proteins such as HRAS, MAP2K1, AKT1, MME, and PTPN1 [44].

### Antipyretic activity

SFJDC could significantly inhibit the body temperature of the dry yeast and LPS-induced pyrexia response [45]. In yeast-induced fever rat model [46, 47], the results showed that SFJDC could significantly reduce body temperature, reduce the levels of prostaglandin E2 (PGE2), TNF-α, IL-1α, IL-1β, IL-6, macrophage inflammatory protein 1 Alpha (MIP-1α), cAMP and the ratio of cAMP/cGMP in serum and hypothalamus. It could also decline the levels of Na^+^, K^+^-ATPase, NOS, NO and the expression of toll-like receptor 4(TLR4), NF-κB p65, IkBα and COX2, enhance the content of endogenous antipyretic medium-arginine vasopressin (AVP) in hypothalamus. SFJDC had a significant antipyretic effect which could reduce heat production, and significantly increase the amount of endogenous antipyretic medium arginine vasopressin in hypothalamus, thus exerting an antipyretic effect.

The summary of pharmacodynamics, mechanism and pharmacodynamic material basis of SFJDC are summarized in Table 1.

## Compatibility rule

The compatibility theory of TCM is the essence of TCM theory, and it is the core content of reflecting the correspondence of prescriptions and syndromes and clinical treatment. Li Ma et al. [48, 49] and Haiyan Bi et al. [50], aiming at the effect of SFJDC on DWRE and CHD, conducted a demerger study from the perspective of functional compatibility, to explore the effects of DWRE, CHD and SFJDC groups on inflammatory response and immune system in rats with acute pneumonia, and to analyze the compatibility rules. The results showed that SFJDC, DWRE and CHD groups could significantly reduce the contents of TNF-α, IFN-γ, IL-1α, IL-1β, IL-2, IL-4, IL-10, NF-κB and COX-2, it can also decrease the colony count and WBC of peripheral blood and alveolar lavage fluid, increase the proportion of eosinophils and reduce the proportion of basophils. The Q values in SFJDC group were beyond 1 on five indicators, which were the contens of TNF-α, IL-1β and IFN-α, the number of WBC and the proportion of eosinophils. Furthermore, SFJDC significantly regulated immune function by reducing the levels of serum IgM, IgG, bradykinin, and CCL2, and the DWRE and CHD groups had significant synergistic effects, which were mainly reflected on indicators including B lymphocyte ratio, NK cell ratio and weight of thymus, spleen, and lung.

Yanqi Han et al. [51] used network pharmacology to predict the potential targets and pathways of 32 compounds selected from DWRE, CHD and ‘Glycyrrhizae radix’ groups of SFJDC. Through data integration analysis, the characteristics and compatibility rules of the prescription were analyzed. The in silico prediction results showed that 32 compounds of SFJDC affected 34 related pathways through 94 target proteins which mainly involved inflammation, lipopolysaccharide and bacterial response, immunoreaction and so on. The three groups not only showed common targets and pathways, but also had their own emphasis on exerting synergistic effects. The analysis found that the CHD group could act on protein targets related to inflammation, lipopolysaccharide and bacterial response, defense response and immunoreaction, indicating that CHD group could directly interfere with the invasion of bacterial lipopolysaccharide, etc., playing a role in detoxification and preventing the development of inflammatory processes. At the same time, it played an auxiliary role in the treatment by stimulating the body’s defense system and enhancing the body’s immunity. The DWRE group could also act on protein targets related to inflammation, bacterial lipopolysaccharide and defense response, and played a therapeutic role in coordination with the CHD group. In addition, the DWRE group could also intervene in the process of sweating and antipyretic through multiple pathways. For example, by indirectly acting on the central fever positive regulatory medium cAMP, inhibiting its production and release, inhibiting the upward movement of the temperature adjustment point, lowering the body temperature, and intervening in the body’s fever process. By interfering with the cholinesterase inhibitor, the acetylcholine released from the cholinergic nerve endings was accumulated, and the M-like effect was enhanced to excite the cholinergic receptor and played a sweating role. Acting on α1 adrenergic receptors mainly distributed in vascular smooth muscle (such as skin, mucosal blood vessels, and some visceral blood vessels) to dilate blood vessels, enhance skin blood circulation, and promote sweating. ‘Glycyrrhizae radix’ group could act on targets related to immune response, glucocorticoid response, lipopolysaccharide response and other processes, and showed auxiliary therapeutic effects by participating in the process of anti-inflammatory and enhancing the body's immunity.

The DWRE, CHD and ‘Glycyrrhizae radix’ groups had common target groups and pathway groups, and each had its own emphasis. The targets involved inflammatory response, immune response, bacterial endotoxin response, defense response, sweating and antipyretic, glucocorticoid response and other links. Each pathway group was connected by a common target, showing multi-target and multi-channel synergy between different components.

## Clinical application

### COVID-19

SFJDC is a recommended drug for the “Influenza Diagnosis and Treatment Protocol” (2020 edition) and the “Diagnosis and Treatment Protocol for COVID-19” (trial 4–10 editions) issued by the National Health Commission. Moreover, SFJDC have been registered in Hong Kong and Macao, China, for the treatment of COVID-19.

SFJDC combined with western medicine treatment in COVID-19 have been gained significant improvement in pneumonia associated symptoms [52]. Studies have shown that compared with antiviral therapy alone, combined with SFJDC in treating COVID-19 patients could significantly shorten the clinical recovery time and improve fatigue and cough symptoms. The strategy of combining SFJDC within the first 8 days after the onset of symptoms could more effectively alleviate the symptoms of patients [28]. SFJDC combined with Arbidol and traditional Chinese and western allopathic medicine to treat common COVID-19, could improve pneumonia regression time, and had better clinical effectiveness with higher white blood cell (WBC) count and lymphocyte percentage, but lower C-reaction protein (CRP) and IL-6 levels without causing serious adverse reactions [7, 53]. Jing Zhang et al. [54] indicated that SFJDC markedly alleviated patients’ symptoms infected with Omicron including a sore throat, coughing, fatigue, and a fever after 7 days of treatment, and the virus negative time was significantly shorter.

### Upper respiratory tract infection

SFJDC has broad-spectrum antibacterial and antiviral effects, which is consistent with the pathogenesis of URI. It is mostly used in treating URI in clinical practice and has a significant effect. Zhaoqing Xi et al. [55] observed the therapeutic effect of SFJDC on the wind heat syndrome of the fever caused by viral URI. In this multi-centered prospective controlled trial, 130 cases (from 5 hospitals) of the fever (wind heat syndrome) caused by viral URI were treated with SFJDC, for 3 days’ treatment and one-day follow-up, to observe the rate of instant antipyretic effect and the antipyretic time. The results showed that the fever was reduced in 39 cases (30.00%) and 118 cases (90.77%) within 4 h and 72 h after medication, respectively. The average antipyretic time was 20.5 h. Therefore, it was considered that SFJDC could treat the fever (wind heat syndrome) caused by the viral URI effectively. Yanling Xu et al. [56] collected 2031 patients with acute URI (wind-heat syndrome) by multi-center and open research method. All patients were given SFJDC, 4 capsules each time, 3 times a day, 30 min after meals. After 3 days of treatment, the curative effect of the disease, the curative effect of TCM syndrome, the curative effect of antipyretic, the curative effect of single symptom or sign were evaluated, and the changes in laboratory indexes and the incidence of adverse reactions during the treatment were observed. It was concluded that SFJDC was safe and effective in treating acute URI (wind-heat syndrome), which was worthy of clinical application.

### Influenza

Influenza is an acute respiratory infection caused by influenza virus, which mainly spreads influenza A and B, leading to an annual epidemic [57]. Jie Niu et al. [58] compared the efficacy of SFJDC and oseltamivir phosphate capsule (OPC) in the treatment of seasonal influenza, including the antipyretic onset time, TCM syndrome efficacy, symptom score changes and safety indexes between the two groups. The results showed that the median antipyretic time of SFJDC was 2 h, which was not different from that of OPC. Both groups could significantly improve the symptoms of influenza, and there was no significant difference. In addition, SFJDC was significantly better than OPC in relieving sore throat and could effectively improve symptoms. Youyue Li et al. [59] observed the therapeutic effect of 754 patients with influenza treated with SFJDC and acyclovir. The results showed that SFJDC combined with acyclovir could significantly reduce the clinical symptoms, and had a good curative effect on influenza, with no obvious adverse reactions, worthy of clinical application. In a prospective pragmatic clinical trial, Hongchao Li et al*.* [60] analyzed the pharmacoeconomics of SFJDC and OPC in patients with influenza-like symptoms. There were 78 and 72 patients in the SFJDC group and OPC group respectively, who were included in the final analyses. The remission rate, response rate and antipyretic effectiveness rate at 48 h or 5 d after treatment were not significantly different between the two groups. The total outpatient expenses and drug costs of SFJDC group were significantly lower than that of the OPC group. The effect of SFJDC and OPC in the treatment of influenza-like symptoms was equivalent. The cost of SFJDC was lower than that of OPC, so SFJDC was more economical in the treatment of influenza-like symptoms.

### Acute exacerbation of chronic obstructive pulmonary disease(AECOPD)

COPD is a lung disease characterized by airflow limitation, which is not completely reversible and progressive. In China, about 99.9 million people, 8.6% of the Chinese population over 20 years old have COPD [61]. AECOPD is defined as an acute exacerbation of respiratory symptoms requiring further treatment [62, 63]. COPD patients have an average of 0.5 to 3.5 acute exacerbations per year [64].

SFJDC is often combined with conventional chemotherapy for the treatment of AECOPD, which can significantly relieve the symptoms of patients and has definite curative effect. Jie Li et al. [65] selected 40 AECOPD patients and randomly divided them into two groups. The control group was given routine treatment, and the experimental group was treated with conventional therapy and SFJDC. The results indicated that the clinical efficacy of the experimental group was 70%, which was significantly higher than that of the control group (55%). The hospital days and the detection rate of RSV, HRV were significantly lower than that of the control group. SFJDC had a good effect on acute exacerbation of chronic obstructive pulmonary disease, which could reduce the average hospital stay and reduce the infection of respiratory syncytial virus and rhinovirus. Ping Wei et al. [66] randomly divided 120 AECOPD patients into experimental and control groups, and the control group was given routine treatment. The results showed that the total effective rate of the experimental group was 93.33%, which was significantly higher than 75.00% of the control group. The clinical related symptoms such as antipyretic time, cough disappearance and rales relief time and the duration of antibiotic use in the treatment group were significantly shortened, while there was no significant difference in the incidence of adverse reactions between the two groups. Xia et al. [67] performed meta-analysis of SFJDC combined with usual care (including antibiotics) in the treatment of AECOPD. Thirteen randomised controlled trials (RCTs) were included, including 1036 patients and 936 inpatients. The results suggested positive effects of SFJDC on significantly reducing treatment failure, duration of hospitalisation, and there was no significant difference in adverse events between SFJDC and control groups. SFJDC may bring more benefits in reducing treatment failure, shortening of hospital stay and improving symptoms. Similarly, Fenglian Tang et al. [68] found that SFJDC could reduce the level of inflammatory factors in patients with AECOPD, alleviate patients’ symptoms and effectively improve patients’ lung function and hemorheology, showing good anti airway remodeling effect and good safety, which could protect lung tissue.

### Community acquired pneumonia

Community acquired pneumonia (CAP) is a common respiratory infectious disease, which refers to the infectious pulmonary parenchymal inflammation outside the hospital, including pneumonia with clear incubation period of pathogen infection and onset within the incubation period after admission [69]. The pathogens include bacteria, viruses, chlamydia and mycoplasma. The specific pathogenic bacteria and drug resistance vary in different regions and change over time [70]. The main clinical symptoms were fever, cough, purulent sputum, hemoptysis, chest pain and other symptoms. In clinical treatment, CAP is mainly based on anti-infection treatment, supplemented by oxygen therapy, atomization, phlegm and other symptomatic treatment. Wenbo Zhou et al. [71] observed 80 CAP patients and randomly divided them into treatment group and control groups, with 40 cases in each group. The control group was given standardized anti-infection treatment, and the treatment group was treated with SFJDC on this basis. The results showed that the disappearance time of clinical symptoms in the treatment group was lower than that of the control group, especially in terms of fever, and no adverse reactions occurred in both groups. Therefore, it was believed that SFJDC combined with moxifloxacin was effective in the treatment of CAP fever patients, and could accelerate the improvement of clinical symptoms and signs. Xiangkun Qu et al. [72] randomly divided 120 patients into two groups. The control group was treated with standardized treatment, and the treatment group was given SFJDC. The results showed that compared with the control group, the recovery time of main clinical symptoms in the treatment group was significantly shortened, and the WBC count, neutrophils, C-reactive protein and platelet hematocrit decreased significantly on the 3rd day after treatment, and returned to normal on the 7th day. The imaging examination showed that the absorption rate of pulmonary inflammatory lesions in the treatment group was higher than that of the control group, and no adverse reactions occurred in the two groups. It could be seen that SFJDC combined with antibiotics in the treatment of CAP could effectively reduce clinical symptoms and accelerate the recovery of infection indicators. Ran Liu et al. [73] collected randomized controlled trials of SFJDC in the treatment of CAP to systematically evaluate the efficacy and safety of SFJDC combined with antibiotics in the treatment of CAP. The results of the meta-analysis showed that compared with antibiotics alone, the curative rate, cough disappearance time, sputum disappearance time, lung rale disappearance time, body temperature stability time, WBC count and C-reactive protein in the combined group were more significant. The efficacy of SFJDC combined with antibiotics in the treatment of CAP was better than that of antibiotics alone.

## Conclusion

This paper systematically summarized the basic and clinical research progress of SFJDC in the treatment of respiratory infectious diseases, providing a theoretical and experimental basis for further in-depth research and clinical promotion and application of SFJDC. According to the modern basic and clinical studies, it has been found that the 94 components that have been identified in SFJDC are mainly iridoids, phenylethanol glycosides, stilbenes, flavonoids, lignans, anthraquinones, triterpenoids, phenolic acids, and alkaloids. Fourty-six exogenous compounds related to SFJDC have been identified in rat plasma, including 27 absorption prototype components and 19 metabolites. Further, the pharmacodynamic material basis of SFJDC have been clarified through high-throughput screening, ‘spectrum-effect’ analysis, network pharmacological prediction, and other methods. Animal and in vitro experiments, network pharmacology, as well as genetic studies have confirmed that SFJDC has antibacterial, antiviral, anti-inflammatory, immunoregulatory, and antipyretic activities, by regulating multi-components, multi-targets, and multi-pathways. DWRE group and CHD group of SFJDC have synergistic effects in anti-inflammation and immune regulation. Moreover, SFJDC has good clinical efficacy in treating COVID-19, URI, influenza, AECOPD, CAP, and other diseases. However, further basic and clinical studies are required to reveal the undiscovered activities, regulatory mechanisms, pharmacodynamic material basis and adverse reactions of SFJDC in the treatment of different respiratory tract infectious diseases.

## Data Availability

Not applicable.

## Abbreviations

SFJDC: Shufeng Jiedu capsule
TCM: Traditional Chinese medicine
URI: Upper respiratory tract infection
AECOPD: Acute exacerbation of chronic obstructive pulmonary disease
COPD: Chronic obstructive pulmonary disease
CAP: Community acquired pneumonia
COVID-19: Corona virus disease 2019
UPLC-Q-TOF–MS/MS: Ultra-performance liquid chromatography quadrupole time-of-fight mass spectrometry
NF-κB: Nuclear factor kappa-B
DWRE: Dispelling wind and relieving exterior
CHD: Learing heat and detoxifying
ANN: Artificial neural network analysis
QKL: Qingkailing granules
HSV: Herpes simplex virus
RSV: Respiratory syncytial virus
CBV: Coxsackievirus B
MAPK: Mitogen-activated protein kinase
BALF: Bronchoalveolar lavage fluid
IL-1β: Interleukin-1 β
IL-18: Interleukin-18
IL-1α: Interleukin-1 α
IL-2: Interleukin-2
IL-4: Interleukin-4
IL-10: Interleukin-10
IFN-α: Interferon-α
NLRP3: NOD-like receptor thermal protein domain associated protein 3
TNF-α: Tumor necrosis factor-α
IFN-γ: Interferon-γ
ACE2: Angiotensin-converting enzyme 2
STAT1: Signal transducer and activator of transcription 1
CCL2: C–C motif chemokine 2
RELA: Transcription factor p65
MAPK1: Mitogen-activated protein kinase 1
ALI: Acute lung injury
GPR18: G protein-coupled receptor 18
A2AAR: A2A adenosine receptor
cAMP: Cyclic adenosine monophosphate
NRF2: Nuclear factor erythroid 2-related factor 2
G-CSF: Granulocyte colony-stimulating factor
GM-CSF: Granulocyte–macrophage colony-stimulating factor
IgA: Immunoglobulin A
IgM: Immunoglobulin M
IgG: Immunoglobulin G
NK: Natural killer cells
GSH-Px: Glutathione peroxidase
MDA: Malondialdehyde
HRAS: GTPase HRas
MAP2K1: Dual specificity mitogen-activated protein kinase
AKT1: RAC-alpha serine/threonine-protein kinase
MME: Neprilysin
PTPN1: Tyrosine-protein phosphatase non-receptor type 1
PGE2: Prostaglandin E2
MIP-1α: Macrophage inflammatory protein 1 Alpha
TLR4: Toll-like receptor 4
AVP: Arginine vasopressin
WBC: White blood cell
CRP: C-reaction protein
OPC: Oseltamivir phosphate capsule
RCTs: Randomised controlled trials
